# Overexpression of Slit2 decreases neuronal excitotoxicity, accelerates glymphatic clearance, and improves cognition in a multiple microinfarcts model

**DOI:** 10.1186/s13041-020-00659-5

**Published:** 2020-10-07

**Authors:** Xiao-fei He, Ge Li, Li-li Li, Ming-yue Li, Feng-yin Liang, Xi Chen, Xi-quan Hu

**Affiliations:** 1 Department of Rehabilitation Medicine, The Third Affiliated Hospital, Sun Yat-sen University, 600 Tianhe Road, Guangzhou, Guangdong China; 2 Guangdong Provincial Key Laboratory of Laboratory Animals, Guangdong Laboratory Animals Monitoring Institute, Guangzhou, 510663 Guangdong China; 3 Department of Neurology, National Key clinical department and Key discipline of Neurology, Guangdong Key Laboratory for diagnosis and Treatment of Major Neurological diseases, The First Affiliated Hospital, Sun Yat-sen University, Guangzhou, 510080 Guangdong China

**Keywords:** Slit2, Microinfarcts, Two-photon imaging, Cognition, Glymphatic clearance

## Abstract

**Background:**

Cerebral microinfarcts (MIs) lead to progressive cognitive impairments in the elderly, and there is currently no effective preventative strategy due to uncertainty about the underlying pathogenic mechanisms. One possibility is the dysfunction of GABAergic transmission and ensuing excitotoxicity. Dysfunction of GABAergic transmission induces excitotoxicity, which contributes to stroke pathology, but the mechanism has kept unknown. The secreted leucine-rich repeat (LRR) family protein slit homologue 2 (Slit2) upregulates GABAergic activity and protects against global cerebral ischemia, but the neuroprotective efficacy of Slit2 against MIs has not been examined.

**Methods:**

Middle-aged Wild type (WT) and *Slit2-Tg* mice were divided into sham and MI treatment groups. MIs were induced in parietal cortex by laser-evoked arteriole occlusion. Spatial memory was then compared between sham and MI groups using the Morris water maze (MWM) task. In addition, neuronal activity, blood brain barrier (BBB) permeability, and glymphatic clearance in peri-infarct areas were compared using two-photon imaging, while GABAergic transmission, microglial activation, neuronal loss, and altered cortical connectivity were compared by immunofluorescent staining or western blotting.

**Results:**

Microinfarcts increased the amplitude and frequency of spontaneous intracellular Ca^2+^ signals, reduced neuronal survival and connectivity within parietal cortex, decreased the number of GABAergic interneurons and expression of vesicular GABA transporter (VGAT), induced neuroinflammation, and impaired both glymphatic clearance and spatial memory. Alternatively, Slit2 overexpression attenuated dysfunctional neuronal Ca^2+^ signaling, protected against neuronal death in the peri-infarct area as well as loss of parietal cortex connectivity, increased GABAergic interneuron number and VGAT expression, attenuated neuroinflammation, and improved both glymphatic clearance and spatial memory.

**Conclusion:**

Our results strongly suggest that overexpression of Slit2 protected against the dysfunction in MIs, which is a potential therapeutic target for cognition impairment in the elderly.

## Introduction

Cerebral microinfarcts (MIs) are wedge-shaped ischemic lesions that result from occlusion of penetrating arterioles [1, 2]. MIs are common in the brains of patients with Alzheimer disease (AD) [3], mild cognitive impairment (MCI) [4], and vascular dementia (VaD) [5], and MI load is associated with the severity of cognitive impairment and dementia in the elderly [6]. Accumulating evidence suggests that MI produces persistent brain inflammation [7] and disorganized axonal structure in both subcortical [8] and cortical tissues [1], thereby expanding regional injury and dysfunction. However, MIs are difficult to detect in living human brain and the extent of these lesions is only revealed by postmortem histological examination [9]. Population aging is currently increasing the global dementia burden, so it is critical to understand the etiology and pathophysiology of microinfarction to aid in the development of effective and safe preventative treatments.

Excessive release of glutamate and concomitant overstimulation of glutamate receptors during and following ischemic stroke (termed excitotoxicity) induces both acute and delayed neuronal death due to excessive calcium influx, oxidative stress, degradation of macromolecules, and activation of apoptotic pathways [10]. Release of gamma-aminobutyric acid (GABA) can counteract glutamate excitotoxicity by inhibiting glutamatergic transmission at presynaptic sites and counteracting glutamate-mediated depolarization of postsynaptic neurons, thereby reducing intracellular calcium deregulation and downstream processes leading to neuronal death [11]. Indeed, augmenting GABAergic transmission can protect against ischemic damage [12, 13]. However, it is uncertain whether a glutamate/GABA imbalance and ensuing excitotoxicity contributes to MI pathology during aging. In addition, research on excitotoxic neuronal damage in cerebral ischemia has focused mainly on the dynamics of excitatory mediators, and much less is known regarding the changes in GABAergic activity [14].

The secreted leucine-rich repeat (LRR) protein slit homologue 2 (Slit2) regulates the migration, development, and axonal path-finding of GABAergic interneurons by stimulating roundabout (Robo) receptors [15]. In addition to regulating GABAergic neuron development and circuit formation, recent studies have also implicated Slit2 signaling in cellular senescence [16] and improved glymphatic clearance [17] as well as inhibition of neuroinflammation and protection against global cerebral ischemia [18]. In the present study, we examined the effects of Slit2 on MIs induced by two-photon irradiation in aging mouse brain.

## Materials and methods

### Animals

Transgenic mice overexpressing human Slit2 (*Slit2-Tg*) [17] were generated as described previously [19] and bred at Guangdong Animal Centre (Guangzhou, China). Wild type (WT) C57BL/6 J mice were also supplied by Guangdong Animal Centre (Guangzhou, China). Mice at 14 months of age were randomly divided into sham and microinfarct (MI) groups. Animals in the sham groups received the same surgery and treatment except for irradiation by a femtosecond laser to induce microinfarct formation.

### Induction of microinfarcts

Anesthesia was induced with 5% isoflurane and maintained by 2.5% isoflurane in oxygen. A 2 × 2 mm^2^ cranial window was then created using a microdrill over the right parietal cortex [20]. Fluorescein isothiocyanate-dextran (FITC-d2000, 1.5% in saline) was injected into the tail vein to image the neurovasculature through a 25× water immersion objective lens positioned over the cranial window. Five penetrating arterioles (PAOs) of 20–25 μm diameter were selected as targets for photobleaching-induced clotting as described [2, 21]. Successful microinfarct induction was confirmed at 24 h post-occlusion by two-photon imaging of target PAOs.

### Morris water maze

Morris water maze testing was performed 1 week after MI modeling as described [22]. Briefly, mice were first examined for spatial learning during five consecutive days of hidden platform training with four trials per day. On day six, the platform was removed and each mouse was tested for spatial memory on a single 60-s probe trial. Swim paths were recorded, and the latency to reach the platform during water maze training as well as the number of crossings over the former platform location (target area) during the probe trial and time spent in the target quadrant during the probe trial were analyzed.

### Two-photon Ca^2+^ imaging

Two weeks after MI induction, the incision was re-opened and the agarose and coverslip over the cranial window were removed. Intracellular Ca^2+^ imaging was performed on the region surrounding the infarct area (Fig. 2a & b) using a two-photon microscope (Leica, Wetzlar, Germany) as described previously [23]. Briefly, peri-ischemic target cells were stained with 10 mM Oregon Green 488 BAPTA-1 AM (OGB-1 AM) diluted 1:10 in standard pipette solution by multicell bolus loading (MCBL) [24]. Sulforhodamine (SR) 101 (1 mg dilution into 4 mL standard pipette solution) was also included to distinguish astrocytes from neurons. The combined staining mixture was injected at a depth of 200–300 μm below the pial surface. Fluorometric Ca^2+^ imaging was performed at 809 nm emission using a two-photon laser scanning microscope. Intracellular Ca^2+^ transients in individual neurons was detected and measured automatically by defining regions of interest (ROIs) in the collected videos and deleting the background fluorescence. Sixty cells located 200 μm below the pial surface (layer 2/3) and surrounding the clotting site were monitored in each animal [25]. Calcium transient amplitudes above the baseline is presented as the relative change in fluorescence (*ΔF*/F), and calcium transient frequency is expressed as the number of fluorescence changes in 1 min (60 s).

### Assessment of the glymphatic efficiency and blood brain barrier (BBB) permeability

Glymphatic clearance and BBB permeability were evaluated as described [17, 26]. Briefly, the animals were anesthetized, the incision was re-opened, FITC-conjugated dextran (40 kDa) dissolved in ACSF was injected into the subarachnoid space via cisterna magna puncture with a microsyringe pump controller, and 200 μL of rhodamine B (70 kDa) was injected intravenously immediately prior to imaging. Images were acquired 5, 15, 30, 45, and 60 min following intra-cisternal FITC-conjugated dextran injection. Changes in FITC-tracer intensity within the paravascular space were quantified to evaluate the glymphatic clearance, changes in rhodamine B intensify in the extravascular compartment were quantified to evaluate the BBB permeability.

### Biotinylated dextran amine (BDA) injection and measurements of axon density

To investigate the effect of microinfarcts on cortical connectivity, the neuroanatomical tracer biotin dextran amine (BDA, MW 10000) was injected into the ipsilateral (exposed/injured) right parietal cortex (0.5 μL of a 5% solution in 0.1 M PBS) and imaged in ipsilateral hippocampus, ipsilateral entorhinal cortex, and contralateral parietal cortex. Animals were perfused through the heart 2 weeks after injection, and brains were fixed, frozen, and coronally sectioned at 10 μm. Six sections spaced 100 μm apart and encompassing the three target areas were stained with Alexa Fluor® 488 Streptavidin, embedded in Fluoroshield™ containing DAPI for nuclear counterstaining and enclosed under a coverslip.

### Histology

Sections were permeabilized with 0.3% Triton and blocked with 10% goat serum for 1 h at room temperature, then incubated overnight at 4 °C with the indicated primary antibody. Immunolabeled sections were then incubated with the indicated secondary antibodies at room temperature in PBS containing 10% normal goat serum for 1 h. Slices were mounted onto slides, embedded in Fluoroshield™ with DAPI, and enclosed under a coverslip. Images were acquired using a Nikon fluorescence microscope or a confocal microscope equipped with a 63× (N.A. 1.25) glycerol immersion objective.

### Western blot analysis

Total protein (20 μg per lane) was separated by SDS-PAGE using 12% precast polyacrylamide gels at 120 V for 90 min. Separated proteins were then transferred to polyvinylidene fluoride membranes at 100 V for 2 h. Membranes were blocked with 5% bovine serum albumin (BSA) at room temperature for 1 h and incubated with the indicated primary antibodies overnight at 4 °C, followed by incubation with anti-rabbit or anti-mouse immunoglobulin G secondary antibody for 1 h.

### Statistical analyses

All data were analyzed by an experimenter blinded to treatment history. All data are expressed as mean ± standard deviation. Immunohistochemical staining and western blotting were analyzed using ImageJ. Mean Slit2 expression levels on western blots were compared by independent-samples t test while other group means were compared by two-way repeated measures ANOVA with Tukey’s post hoc tests for multiple comparisons. All statistical analyses were conducted using SPSS 19.0. A *P* < 0.05 (two tailed) was considered statistically significant for all tests.

## Results

### Slit2 was overexpressed in neurons and astrocytes but not in microglia/ macrophage of transgenic mice

Western blotting was performed to confirm expression of the human Slit2 transgene protein in transgenic (Tg) mouse brain, and indeed expression was significantly elevated in *Slit2-Tg* mice compared to WT mice (*P* < 0.001) (Supplementary Fig. 1a). Co-immunofluorescence staining using anti-Flag for detection of Slit2 and cell type-specific antibodies revealed overexpression in neurons and astrocytes but not in microglia/macrophage (Supplementary Fig. 1b-d).

### Overexpression of Slit2 improved Morris water maze performance in mice with parietal microinfarcts

**Fig. 1:**
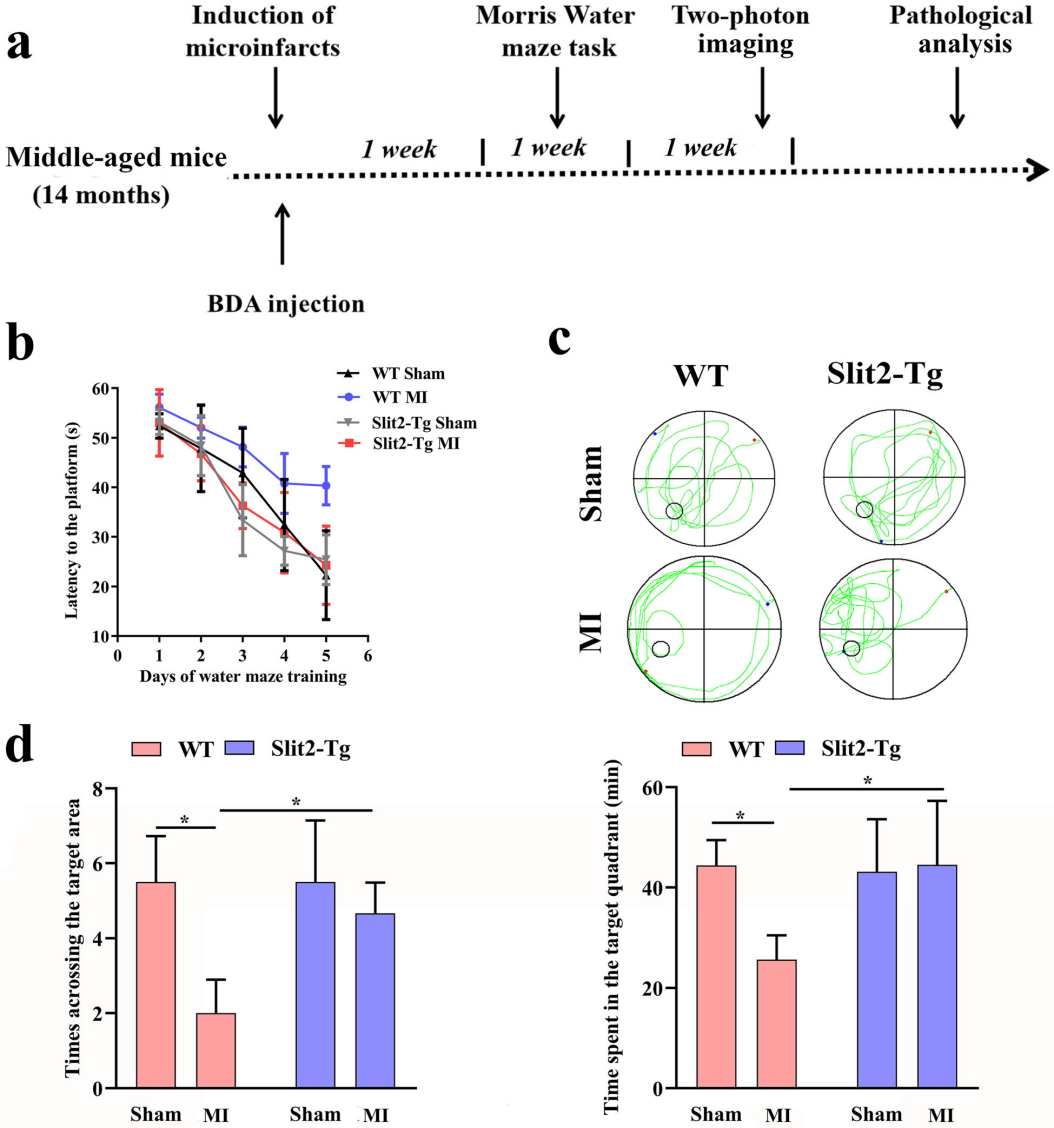
Overexpression of Slit2 improves spatial learning and memory deficits caused by cerebral microinfarct (CMI) induction. **a** Experimental timeline of BDA injection and Morris water maze (MWM) testing, Two-photon imaging and pathological analysis. **b** Latencies to the hidden platform (escape latencies) during training trials by wild type (WT) sham, WT cerebral microinfarct (MI), *Slit2-Tg* sham, and *Slit2-Tg* MI groups. **c** Swim paths during the probe trial. **d** Number of target area crossings and time spent in the target quadrant during the probe trial. Each dataset is expressed as mean ± SD. ^*^P ≤ 0.05; ^**^P ≤ 0.01; ^***^P ≤ 0.001; ^****^P ≤ 0.0001. *n* = 6 mice

The posterior parietal cortex (PPC) is a multimodal association area involved in spatial navigation as evidenced by performance deficits in the Morris water maze (MWM) following PPC lesions [27]. To explore the protective efficacy of Slit2 against cognitive dysfunction due to PPC microinfarcts in middle-aged (14-month-old) mice, we compared MWM performance between WT and *Slit2-Tg* mice following sham treatment or MI induction (Fig. 1a). As shown in Fig. 1b, on days 4 and 5 of training, there were no significant differences in escape latencies between WT sham and *Slit2-Tg* sham groups (Both *P* > 0.05), but escape latencies were significantly shorter in *Slit2-Tg* MI mice than WT MI mice (*P* < 0.05 and *P* < 0.001, respectively). These findings suggest that Slit2 overexpression protects against spatial learning impairment following MI induction.

During the probe trial (Fig. 1c & d), the number of platform crossings was significantly reduced by MI induction in WT mice (MI vs. Sham, *P* < 0.05), but not in *Slit2-Tg* mice (MI vs. Sham, *P* > 0.05); moreover, the number of platform crossing was lower in the WT MI group compared to the *Slit2-Tg* MI group (*P <* 0.05). Similarly, the target quadrant time was significantly reduced by MI induction in WT mice (*P* < 0.05 vs. WT sham mice), but not in *Slit2-Tg* mice (*P* > 0.05), and quadrant time was shorter in WT MI mice than *Slit2-Tg* MI mice (*P* < 0.05). These results suggest that microinfarcts cause spatial memory deficits that can be improved by Slit2 overexpression.

### Overexpression of Slit2 inhibited neuronal hyperactivation in the peri-infarct area

**Fig. 2:**
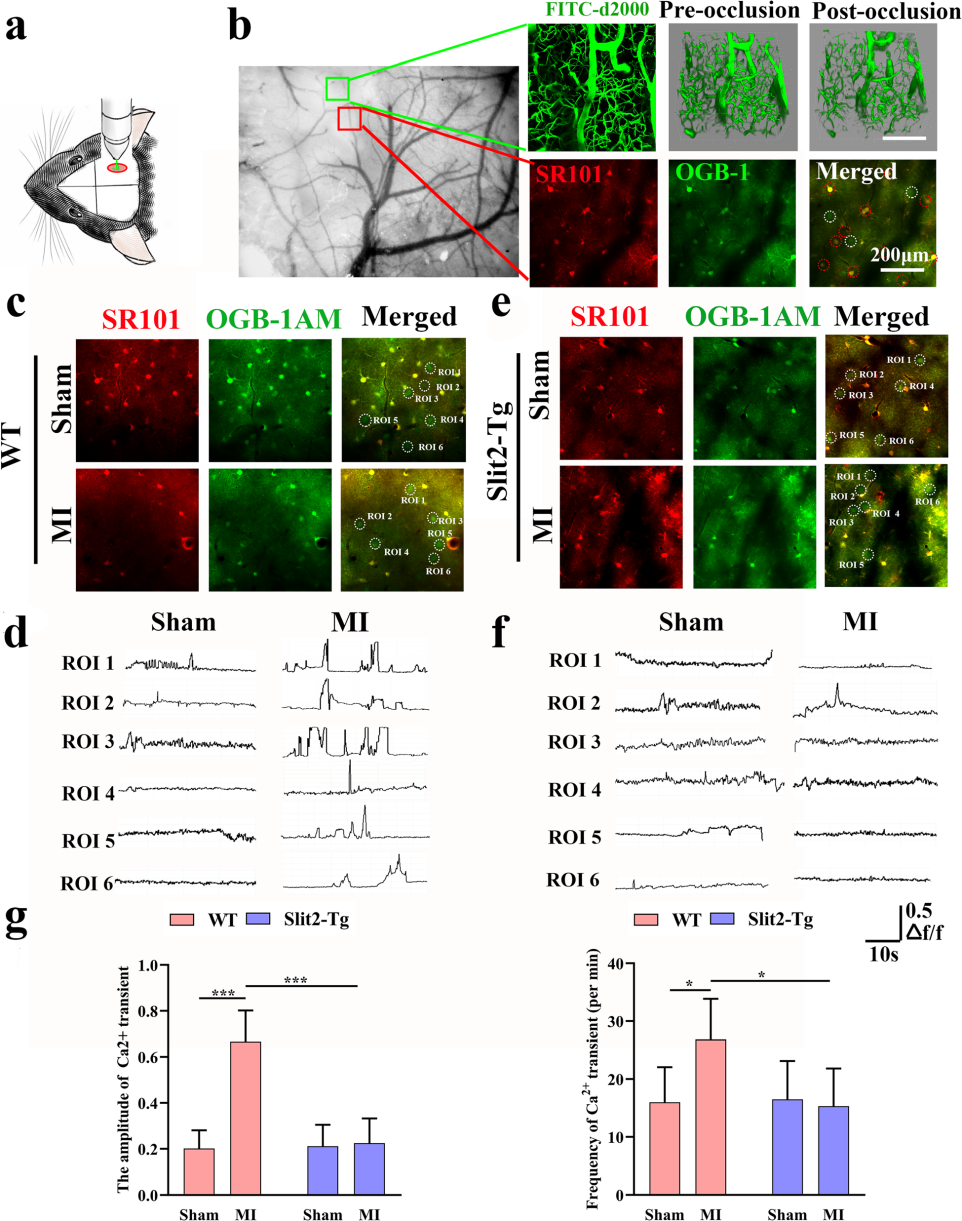
Overexpression of Slit2 reduces spontaneous Ca^2+^ signal amplitude and frequency in the peri-infarct region. **a**, **b** Diagram showing the site of microinfarct (MI) induction and two-photon Ca^2+^ imaging. **c** Representative Ca^2+^ images in the peri-infarct area of a WT mouse. White circles show six representative neurons monitored for Ca^2+^ signals over 60 s. **d** Representative Ca^2+^ signals in the six representative neurons (regions of interest, ROIs) indicated in **c**. **e** Representative Ca^2+^ images of the peri-infarct area in a *Slit2-Tg* mouse. White circles show the six representative neurons monitored for 60 s. **f** Representative Ca^2+^ signals in the OGA-1 AM-positive neurons (ROIs) indicated in E. **g** Comparisons of Ca^2+^ signals between sham and MI groups of WT and *Slit2-Tg* mice. Each dataset is expressed as mean ± SD. ^*^P ≤ 0.05; ^**^P ≤ 0.01; ^***^P ≤ 0.001; ^****^P ≤ 0.0001. *n* = 6 mice

Two-photon Ca^2+^ imaging in the peri-infarct area was performed 2 weeks after MI induction to assess effects on local excitatory/inhibitory balance (Fig. 2a & b). The amplitude of spontaneous intracellular Ca^2+^ transients was significantly enhanced by MI in WT mice (*P* < 0.001, MI vs. sham mice) (Fig. 2c, d, g), but not in *Slit2-Tg* mice (*P* > 0.05) (Fig. 2e, f, g). In addition, MI increased the frequency of these Ca^2+^ transients in WT mice (*P* < 0.05, MI vs. sham mice) (Fig. 2c, d, g), but not *Slit2-Tg* mice (*P* > 0.05) (Fig. 2e, f, g). Collectively, these findings indicate that Slit2 overexpression suppresses neuronal Ca^2+^ transients in the peri-infarct area, possibly by reducing neuronal excitability.

### Overexpression of Slit2 protected against axonal damage after multiple cortical microinfarct induction

**Fig. 3:**
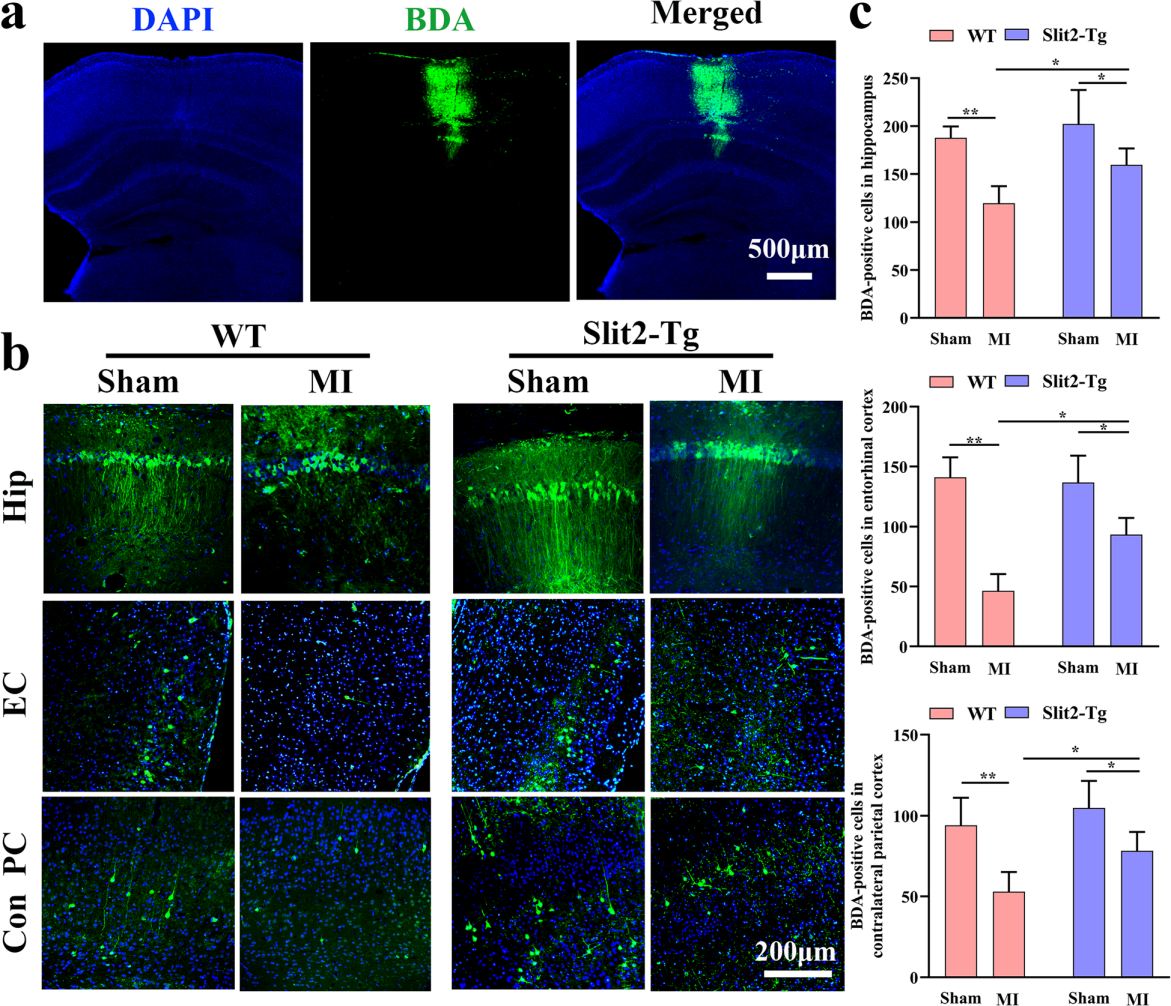
Overexpression of Slit2 protects against dysfunctional axonal connectivity induced by MIs. **a** Representative image showing BDA injection (10×). **b** Images of BDA-positive neurons and axons in ipsilateral hippocampus, ipsilateral entorhinal cortex, and contralateral parietal cortex. **c** Comparison of BDA-positive cells in ipsilateral hippocampus, ipsilateral entorhinal cortex, and contralateral parietal cortex among sham and MI groups of WT and *Slit2-Tg* mice (20×, 1 field/slice, 5 slices per mouse). Each dataset is expressed as mean ± SD. ^*^P ≤ 0.05; ^**^P ≤ 0.01; ^***^P ≤ 0.001; ^****^P ≤ 0.0001. *n* = 6 mice

The anatomical tracer BDA was injected into the right (lesioned) parietal cortex (Fig. 3a) and transport to ipsilateral hippocampus, ipsilateral entorhinal cortex, and contralateral parietal cortex was examined as an index of connectivity. The BDA-positive cell number was significantly higher in ipsilateral hippocampus of *Slit2-Tg* MI mice compared to WT MI mice (*P* < 0.05) (Fig. 3b & c). Similarly, BDA-positive cell number was significantly higher in ipsilateral entorhinal cortex of *Slit2-Tg* MI mice compared to WT MI mice (*P* < 0.05) (Fig. 3b & c). Finally, BDA-positive cell number was also significantly higher in contralateral parietal cortex of *Slit2-Tg* MI mice compared to WT MI mice (*P* < 0.05) (Fig. 3b & c). These results indicate that MIs can disrupt both intrahemispheric and interhemispheric cortical projections and that Slit2 overexpression can preserve these projections following MI induction.

### Overexpression of Slit2 increased GABAergic interneuron number and VGATs in the peri-infarct area

**Fig. 4:**
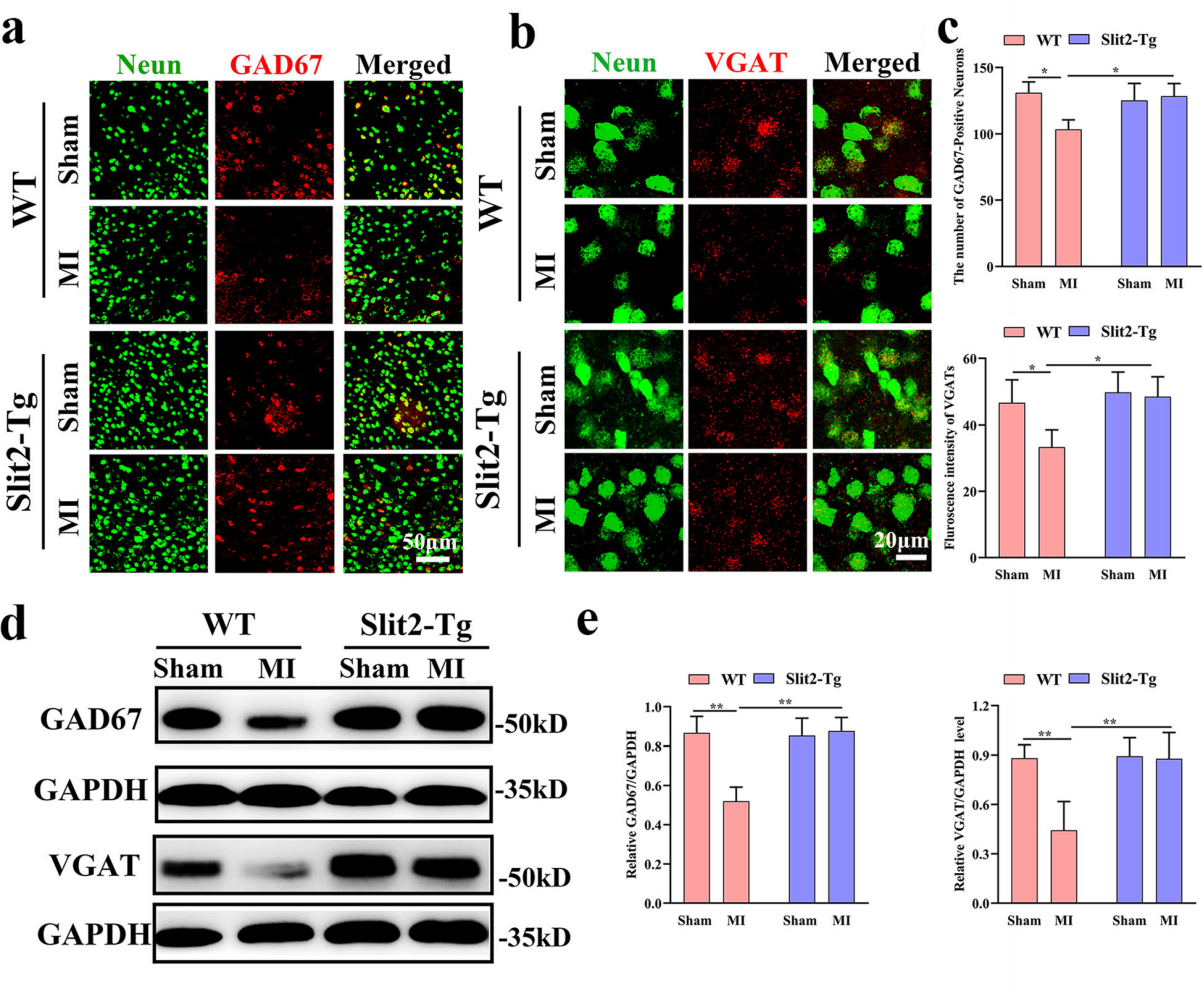
Overexpression of Slit2 increases peri-infarct GABAergic activity. **a** Immunoexpression of the GABAergic interneuron marker GAD67 and the neuronal marker Neun in peri-infarct areas (40 ×). **b** Immunofluorescence staining of VGAT in peri-infarct areas (63 ×, zoomed in 3). **c** Comparison of GABAergic interneuron number and mean VGAT immunofluorescence intensity among sham and MI groups of WT and *Slit2-Tg* mice. **d** Chemiluminescence imaging of GAD67 and GAPDH, or VGAT and GAPDH in the peri-infarct area. **e** Comparison of GAD67/GAPDH and VGAT/GAPDH expression ratios in the peri-infarct area among sham and MI groups of WT and *Slit2-Tg* mice. Each dataset is expressed as mean ± SD. ^*^P ≤ 0.05; ^**^P ≤ 0.01; ^***^P ≤ 0.001; ^****^P ≤ 0.0001. *n* = 6 mice

To directly examine if Slit2 overexpression regulates excitatory/inhibitory balance in peri-infarct areas by increasing the number of GABAergic neurons, we measured the immunoexpression of the GABAergic interneuron markers GAD67 (Fig. 4a) and vesicular GABA transporter (VGAT) (Fig. 4b). The number of GAD67-positive neurons surrounding the infarct area was significantly lower in WT MI mice compared to the WT sham mice (*P* < 0.05), but did not differ between *Slit2-Tg* MI and sham mice (*P* > 0.05) (Fig. 4c). In accord with this result, VGAT intensity was significantly lower in WT MI mice than sham mice (*P* < 0.05), but did not differ between *Slit2-Tg* MI and sham mice (*P* > 0.05) (Fig. 4c). Finally, we performed western blotting to verify the expression levels of GAD67 and VGAT in peri-infarct areas (Fig. 4d & e). GAD67 expression was significantly lower in WT MI mice compared to WT sham mice (*P* < 0.01), but did not differ between *Slit2-Tg* MI and sham mice (*P* > 0.05). Furthermore, GAD67 expression was significantly lower in WT MI mice than *Slit2-Tg* MI mice (*P* < 0.01). VGAT expression was also significantly lower in WT MI mice than WT sham mice (*P* < 0.01), but did not differ between *Slit2-Tg* MI and sham mice (*P* > 0.05). Moreover, VGAT expression was significantly lower in WT MI mice than *Slit2-Tg* MI mice (*P* < 0.01).

### Overexpression of Slit2 attenuated peri-infarct neuroinflammation and protected against local neuronal loss

**Fig. 5:**
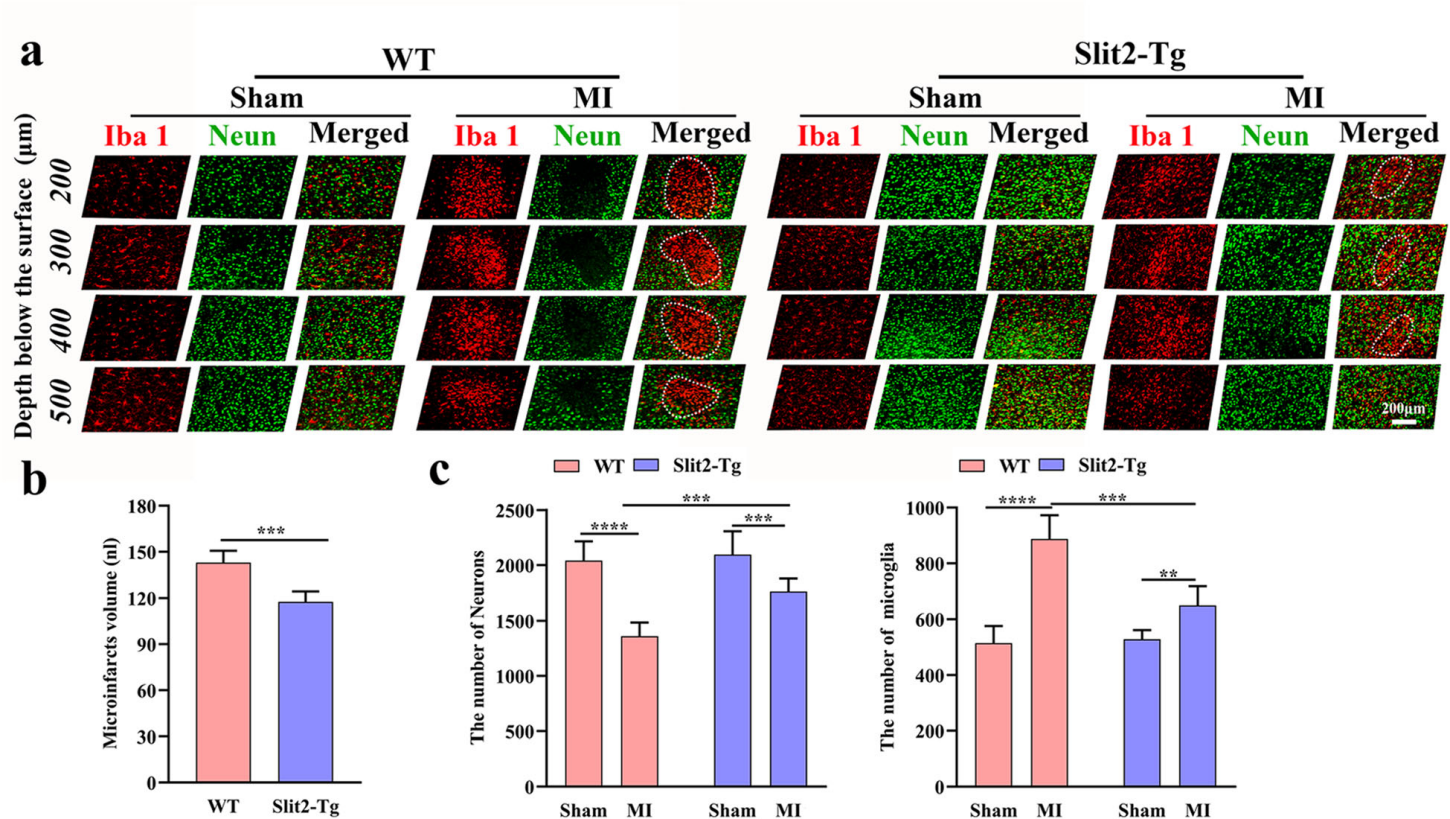
Overexpression of Slit2 reduces peri-infarct neuronal loss and microglia/macrophage activation. **a** Immunofluorescence staining of neurons and microglia/macrophage (20×). **b** Comparison of microinfarct volumes between WT MI and Slit2-Tg MI groups. **c** Comparisons of neuron and microglia/macrophage numbers among WT sham, WT MI, *Slit2-Tg* sham, and *Slit2-Tg* MI groups (20×, 1 field/slice, 5 slices per mouse). Each dataset is expressed as mean ± SD. ^*^P ≤ 0.05; ^**^P ≤ 0.01; ^***^P ≤ 0.001; ^****^P ≤ 0.0001. *n* = 6 mice

Microinfarct volume was calculated by the method of Luo [2], it was significantly lower in *Slit2-Tg* MI mice than WT MI mice (*P* < 0.001) (Fig. 5a & b). Neuronal numbers were significantly reduced in MI groups compared to corresponding sham groups, both for WT mice (*P* < 0.0001) and *Slit2-Tg* mice (*P* < 0.001) (Fig. 5a & c), but number was significantly greater in *Slit2-Tg* MI mice than WT MI mice (*P* < 0.001). Conversely, the numbers of microglia/macrophage were significantly greater in both WT MI mice (*P* < 0.0001) and *Slit2-Tg* MI mice (*P* < 0.01) compared to the corresponding sham controls, suggesting local neuroinflammation, but were significantly lower in *Slit2-Tg* MI mice than WT MI mice (*P* < 0.001). Furthermore, Slit2 overexpression appears to protect neurons for MI-induced degeneration, possibly by quelling the ensuing neuroinflammatory response.

### Overexpression of Slit2 improved glymphatic clearance and protected against the BBB dysfunction

**Fig. 6:**
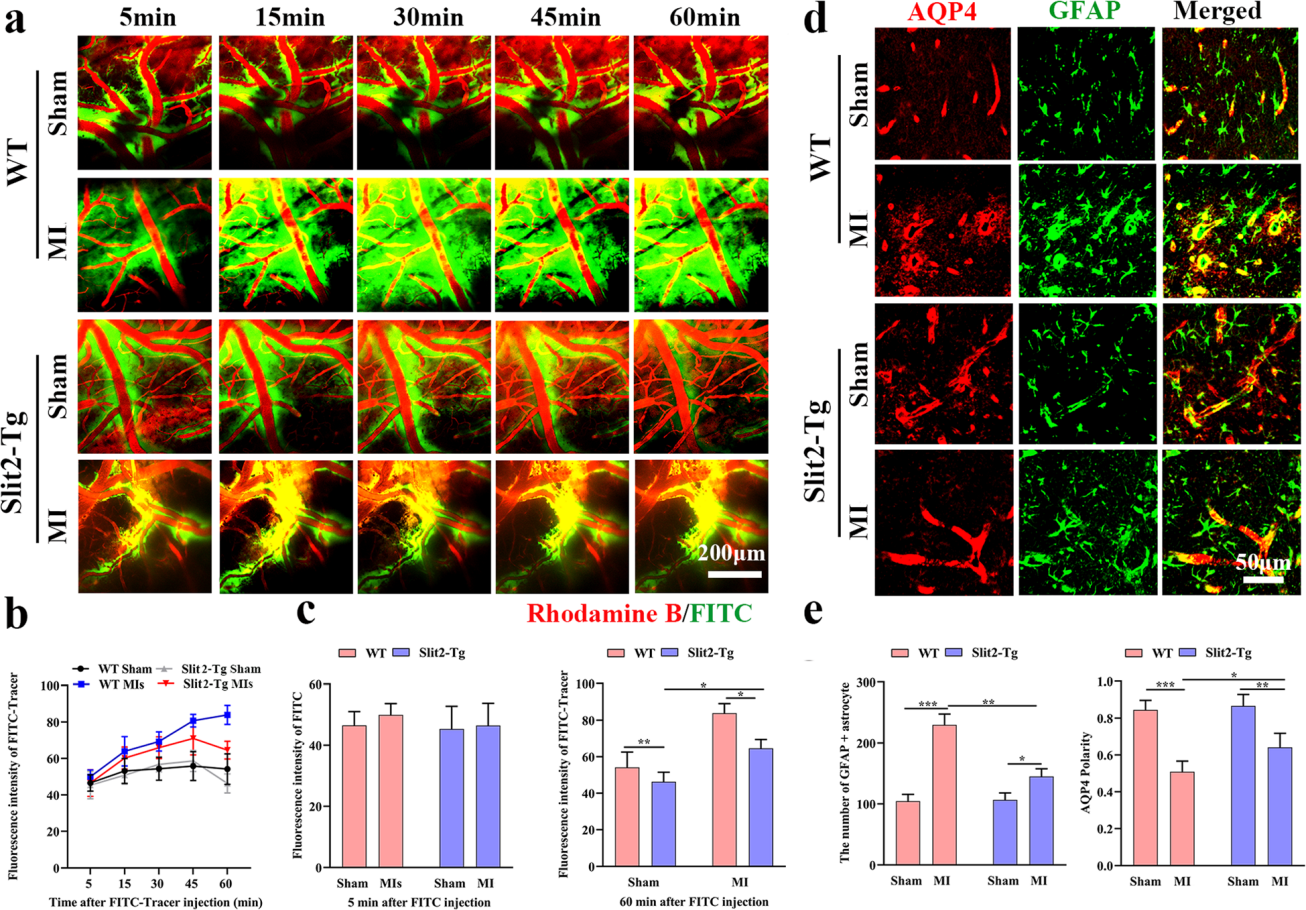
Overexpression of Slit2 improves paravascular clearance in the peri-infarct area. **a** Representative *xyz* overlaid images of the cortical vascular (Rhodamine B: red color) and paravascular spaces (FITC-dextran: green color) in the peri-infarct area at 5, 15, 30, 45, and 60 min after FITC-dextran injection (25 × objective). **b** Linear analysis of the FITC-dextran fluorescence emission intensity in the paravascular space. **c** Histograms of FITC-dextran fluorescence intensity in the paravascular space at 5 and 60 min after cisterna magna injection among WT sham, WT MI, *Slit2-Tg* sham, and *Slit2-Tg* MI groups. **d** Immunofluorescence staining of AQP4 and the astrocyte marker GFAP (25 × objective, magnified by 3). **e** Comparison of GFAP+ astrocyte numbers and AQP4 polarity among WT sham, WT MI, *Slit2-Tg* sham, and *Slit2-Tg* MI groups. Each dataset is expressed as mean ± SD. ^*^P ≤ 0.05; ^**^P ≤ 0.01; ^***^P ≤ 0.001; ^****^P ≤ 0.0001. *n* = 6 mice

As shown in Fig. 6a, FITC-dextran tracer moved along the paravascular space and rapidly entered the interstitium of the parenchyma following intra-cisternal injection. The FITC intensities at 5 min did not differ significantly among WT sham, WT MI, *Slit2-Tg* sham, and *Slit2-Tg* MI groups (Fig. 6b & c). In all groups, parenchymal/perivascular FITC-dextran fluorescence intensity gradually increased over the first 45 min after injection. Thereafter, intensity continued to increase in the WT MI group, indicating dysfunction of glymphatic clearance, but decreased in the other three groups. At 60 min after FITC-dextran injection (Fig. 6b & c), FITC intensities were significantly higher in both WT MI mice versus sham mice (*P* < 0.01) and *Slit2-Tg* MI mice versus sham mice (*P* < 0.05). Further, FITC intensity was significantly lower in *Slit2-Tg* MI mice compared to WT MI mice (*P* < 0.05) (Fig. 6c), suggesting that Slit2 overexpression sustained glymphatic clearance following MI induction.

Reactive astrocyte numbers were significantly greater in peri-infarct areas of MI groups compared to corresponding sham groups, both for WT (*P* < 0.001) and *Slit2-Tg* mice (*P* < 0.05) (Fig. 6d & e). Mean reactive astrocyte number was also significantly higher in WT MI mice compared to *Slit2-Tg* MI mice (*P* < 0.01), suggesting that Slit2 overexpression can suppress astrocyte reactivity in response to MI induction.

The normal polarity of AQP4 surface distribution on astrocytes (endfeet > soma) was disrupted (reduced) in MI groups compared to corresponding sham groups, both for WT mice (*P* < 0.001) and *Slit2-Tg* mice (*P* < 0.01). However, polarity was still significantly greater in *Slit2-Tg* MI mice compared to WT MI mice (*P* < 0.05), indicating that while MI induction disrupted astrocytic AQP4 distribution, Slit2 overexpression better sustained the normal polarity.

To investigate the effect of MIs on BBB integrity and potential protection by Slit2 overexpression, we measured the rate of rhodamine B permeation into the extravascular compartment following venous injection. Rhodamine B begun to permeate into the extravascular compartment at 15 min post-injection and the fluorescence intensity gradually increased thereafter in all four groups (Supplementary Fig. 2a-c). There were no significant differences in rhodamine B intensity among these four groups at 5 min post-injection (*P* > 0.05). At 60 min after injection, however, rhodamine B intensity was significantly greater in the MI groups compared to corresponding sham controls, both for WT (*P* < 0.001) and *Slit2-Tg* mice (*P* < 0.05), suggesting that MIs damaged the BBB. Nonetheless, extravascular rhodamine B fluorescence at 60 min post-injection was significantly lower in *Slit2-Tg* MI mice than WT MI mice (*P* < 0.001), suggesting that Slit2 overexpression maintains BBB integrity.

## Discussion

Increase of neuronal Ca2+ activity has been associated with the neuronal excitotoxicity, which is one of the primary pathogenic mechanisms for cell death in stroke [28, 29], so inhibiting Ca2+ hyperactivity or excitotoxicity is expected to protect against ensuing brain damage. We here described that neuronal Ca2+ hyperactivity contributed to the neuron death in microinfarcts. We further demonstrated that overexpression of Slit2 inhibited the neuronal Ca2+ hyperactivity and protected against the neuron death. This inhibition was accompanied by an increase of GAD67 positive cells and VGAT expressions. GAD67 is a marker for GABAergic neurons and VGAT is a marker for vesicular GABA transporter that release GABA [30, 31]. GABA is the primary inhibitory neurotransmitter in the mammalian brain, which has been demonstrated to inhibit the Ca2+ overload [32], and protect neurons from excitotoxicity during stroke [33].

Slit2 was reported to be overexpressed in these Tg mice throughout life [17, 19, 34], which has been shown to promote axonal plasticity in developing cortical cells [35, 36]. Consistently, we demonstrated that Slit2 overexpression promoted neuronal plasticity after cortical microinfarct induction. This action may be also related to GABAergic interneurons because GABA-mediated inhibition is a critical modulator of cortical remapping, which is required for functional recovery after stroke [37]. It is not clear how Slit2 modulates GABAergic function in stroke. One potential mechanism may be related to the development of interneuron populations and GABAergic function in cerebral cortex [38], [39], Slit2 signaling protects against interneuronal loss and reduced the excitotoxic sequela [38]. Thus, it is likely that Slit2 modulates GABAergic function to inhibit neuronal Ca + overloading and enhance plasticity. Further study need to be done to explore how Slit2 signaling regulates neuronal plasticity via GABAergic transmission.

In addition to inhibition of calcium overload and enhancement of neuronal plasticity, we found that overexpression of Slit2 improved the glymphatic system and BBB integrity after microinfarcts induction, which was consistent with previous study [17]. This protection may be associated with its anti-inflammatory property [18]. Neuroinflammation is the critical pathological feature in microstroke [40–42], it impairs the glymphatic clearance by disrupting the distribution of astroglial AQP4 polarity from the endfeet to the soma [43], and it increases the BBB permeability by changing the tight junction proteins [44]. Inhibiting the neuroinflammation improves the paravascular clearance and BBB integrity [45]. However, Han [19] showed that Slit2 increased the permeability of brain vessels to large molecules. There are two possible reasons for this inconsistency. Firstly, the mice we used were at age of 14 months, they were much older than Han’s study, the cellular and molecular elements of BBB are changing during aging [46]. Besides, the BBB permeability was detected by measuring the leakage of rhodamine B within 60 min after injection in our study, but Han [19] detected the amount of Evans blue dye 20 min after injection to test the BBB integrity. There is a problem for Han’s methodology, the residual Evans blue dye in brain capillaries might bind to plasma proteins and spectral shifts [47].

It is worthy of note that parietal cortex contributes to route learning using proximal salient cues in the water maze task [48]. Thigmotaxis (swimming alone the tub edge) is indicative of spatial learning failure [49] and we observed thigmotaxis in WT MIs mice, but not *Slit2-Tg* MI mice, suggesting that Slit2 overexpression protected against the spatial learning impairment induced by parietal microinfarcts. There is a limitation in our study, we used *Slit2-Tg* mice that overexpressing human Slit2 rather than mouse Slit2 because of the availability. Numerous studies have demonstrated that these *Slit2-Tg* mice could be used to study the function of Slit2 [50–52]. Slit2 gene has over 90% homology among different species [53], and human *Slit2* expression is efficiently and non-selectively [50] binding to Robo Receptors in vertebrate species [54].

In summary, we demonstrated that Slit2 signaling has multiple functions, it can increase the GABAergic interneurons and VGAT expression in peri-infarct regions, thereby inhibiting the neuronal calcium overload and local neuroinflammation. Furthermore, Slit2 overexpression protected against glymphatic system and BBB dysfunction, attenuated local neuronal loss, and ultimately prevented cognitive decline induced by parietal microinfarcts.

## Supplementary information

**Additional file 1: Figure S1.** Slit2 is overexpressed in cortical neurons and astrocytes, but not microglia of *Slit2-Tg* mice. A. Western blotting analysis of human (h)Slit2 expression. B. Immunofluorescence analysis of hSlit2 expression in neurons using Flag-tag and Neun antibodies (63×). C. Immunofluorescence analysis of hSlit2 expression in microglia using Flag-tag and Iba 1 antibodies (63×). D. Immunofluorescence analysis of hSlit2 expression in astrocyte using Flag-tag and GFAP antibodies (63×). **Figure S2.** Overexpression of Slit2 protects against blood brain barrier (BBB) dysfunction in the peri -infarct area. A. Representative *xyz* overlaid images of the cortical vasculature in the peri-infarct area at 5, 15, 30, 45, and 60 min after Rhodamine B injection (25 × objective). B. Linear analysis of the Rhodamine B fluorescence intensity in the extracellular compartment. C. Histograms of Rhodamine B fluorescence intensity in the extracellular compartment at 5 and 60 min post-injection among WT sham, WT MI, S*lit2-Tg* sham, and *Slit2-Tg* MI groups. Each dataset is expressed as mean ± SD. ^*^P ≤ 0.05; ^**^P ≤ 0.01; ^***^P ≤ 0.001; ^****^P ≤ 0.0001. *n* = 6 mice.

## Data Availability

Data openly available in a public repository that issues datasets with DOIs.

## Abbreviations

MWM: Morris water maze
Slit2: Secreted LRR protein slit homologue 2
PBS: Phosphate-buffered saline
GABA: γ-aminobutyric acid
GAD67: Glutamic acid decarboxylase 67
VGAT: Vesicular GABA Transporter
MIs: Microinfarcts
AQP4: Aquaporin
BBB: Blood brain barrier (BBB)
AD: Alzheimer disease
FITC: Fluorescein isothiocyanate-dextran
PPC: Posterior parietal cortex
BDA: Biotinylated dextran amine
