# Supplementary material for: Overexpression of Slit2 decreases neuronal excitotoxicity, accelerates glymphatic clearance, and improves cognition in a multiple microinfarcts model
Source: Mol Brain. 2020 Oct 7;13:135. doi: 10.1186/s13041-020-00659-5 (PMC7542754; doi:10.1186/s13041-020-00659-5)
Supplement: Supplementary file 1 — Additional file 1: Figure S1. Slit2 is overexpressed in cortical neurons and astrocytes, but not microglia of Slit2-Tg mice. A. Western blotting analysis of human (h)Slit2 expression. B. Immunofluorescence analysis of hSlit2 expression in neurons using Flag-tag and Neun antibodies (63×). C. Immunofluorescence analysis of hSlit2 expression in microglia using Flag-tag and Iba 1 antibodies (63×). D. Immunofluorescence analysis of hSlit2 expression in astrocyte using Flag-tag and GFAP antibodies (63×). Figure S2. Overexpression of Slit2 protects against blood brain barrier (BBB) dysfunction in the peri -infarct area. A. Representative xyz overlaid images of the cortical vasculature in the peri-infarct area at 5, 15, 30, 45, and 60 min after Rhodamine B injection (25 × objective). B. Linear analysis of the Rhodamine B fluorescence intensity in the extracellular compartment. C. Histograms of Rhodamine B fluorescence intensity in the extracellular compartment at 5 and 60 min post-injection among WT sham, WT MI, Slit2-Tg sham, and Slit2-Tg MI groups. Each dataset is expressed as mean ± SD. *P ≤ 0.05; **P ≤ 0.01; ***P ≤ 0.001; ****P ≤ 0.0001. n = 6 mice. [file 13041_2020_659_MOESM1_ESM.doc]

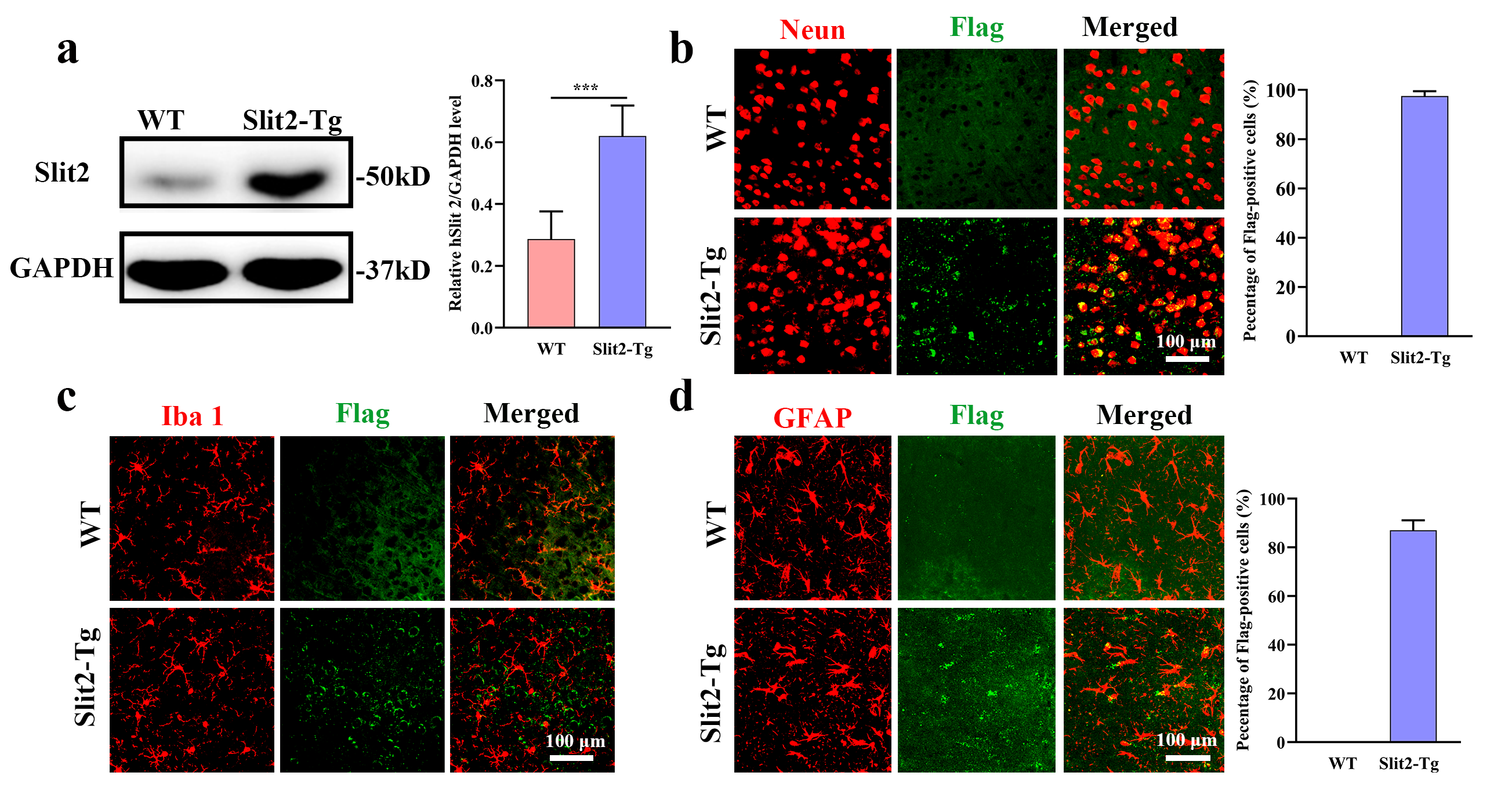


Supplementary Figure 1. Slit2 is overexpressed in cortical neurons and astrocytes, but not microglia of *Slit2-Tg* mice. A. Western blotting analysis of human (h)Slit2 expression. B. Immunofluorescence analysis of hSlit2 expression in neurons using Flag-tag and Neun antibodies (63×). C. Immunofluorescence analysis of hSlit2 expression in microglia using Flag-tag and Iba 1 antibodies (63×). D. Immunofluorescence analysis of hSlit2 expression in astrocyte using Flag-tag and GFAP antibodies (63×).


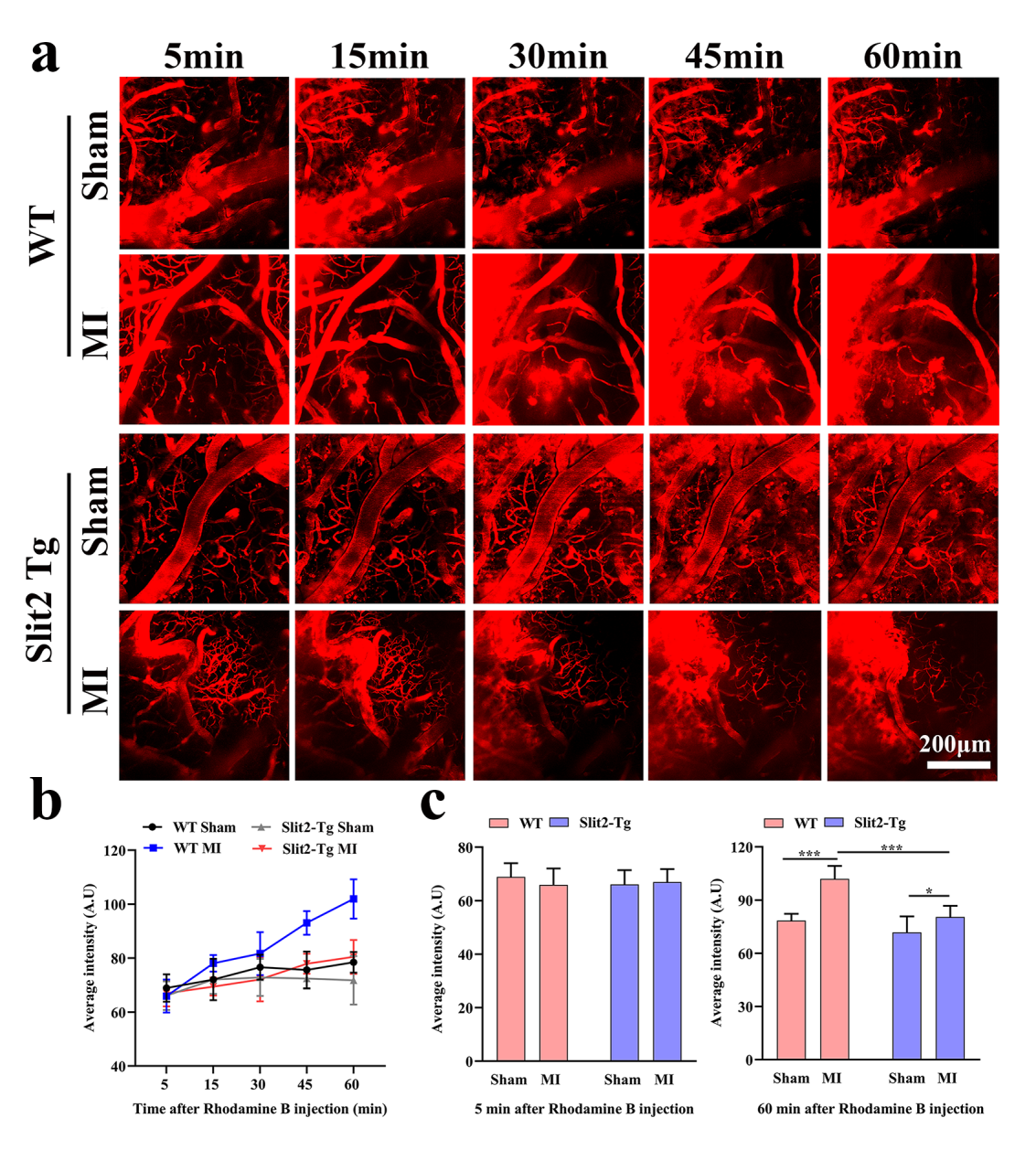


Supplementary Figure 2. Overexpression of Slit2 protects against [blood](../../../../C:/Program%20Files%20(x86)/Youdao/Dict/8.8.1.0/resultui/html/index.html#/javascript:;) [brain](../../../../C:/Program%20Files%20(x86)/Youdao/Dict/8.8.1.0/resultui/html/index.html#/javascript:;) [barrier](../../../../C:/Program%20Files%20(x86)/Youdao/Dict/8.8.1.0/resultui/html/index.html#/javascript:;) (BBB) dysfunction in the peri -infarct area. A. Representative *xyz* overlaid images of the cortical vasculature in the peri-infarct area at 5, 15, 30, 45, and 60 min after Rhodamine B injection (25 × objective). B. Linear analysis of the Rhodamine B fluorescence intensity in the extracellular compartment. C. Histograms of Rhodamine B fluorescence intensity in the extracellular compartment at 5 and 60 min post-injection among WT sham, WT MI, S*lit2-Tg* sham, and *Slit2-Tg* MI groups. Each dataset is expressed as mean ± SD. ^*^*P* ≤ 0.05; ^**^*P* ≤ 0.01; ^***^*P* ≤ 0.001; ^****^*P* ≤ 0.0001. n=6 mice.
